# Supplementary material for: In Vitro Mineralisation of Tissue-Engineered Cartilage Reduces Endothelial Cell Migration, Proliferation and Tube Formation
Source: Cells. 2023 Apr 20;12(8):1202. doi: 10.3390/cells12081202 (PMC10136546; doi:10.3390/cells12081202)
Supplement: Supplementary file 1 [file cells-12-01202-s001.zip › Supplementary figures.pdf]

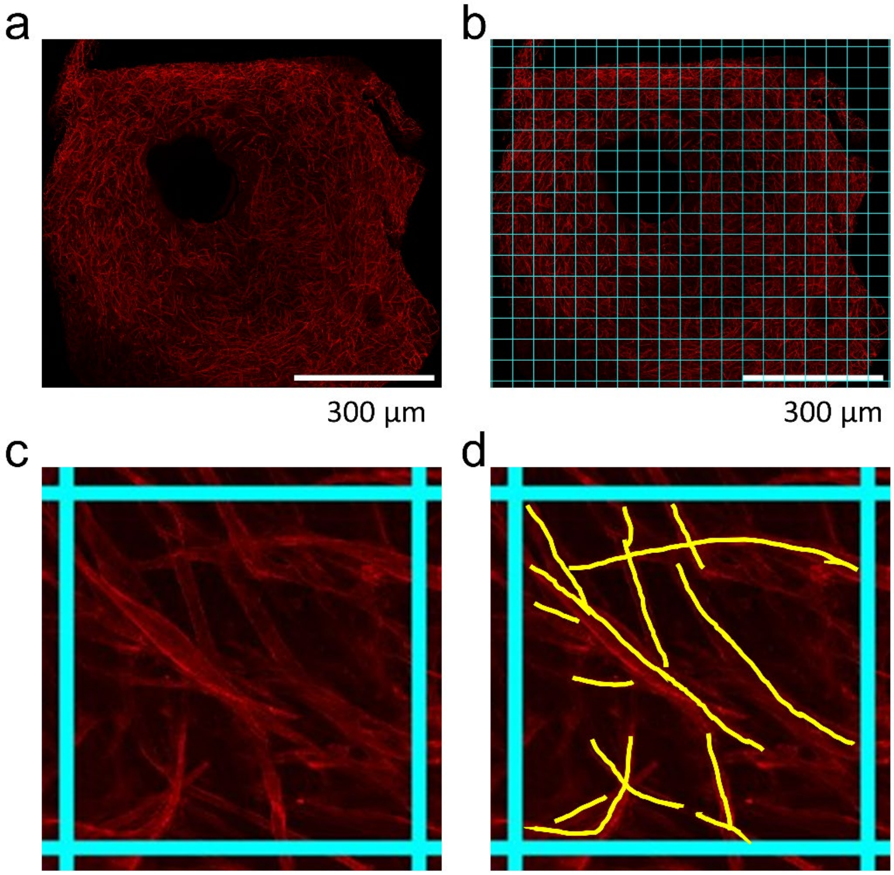

Figure S1. Schematics of the quantification of the vessel length density (VLD) in 3D tube formation assay. To evaluate 3D vessel network density, (**a**) the whole image of the stained vessel network is acquired. (**b**) Next, the ImageJ software is used to set multiple squares (200000 µm^2 per square) in the image, (**c**) and 10 squares are randomly selected from the whole image (excluding blank areas). (**d**) For each square, the tube structures are drawn with freehand line in ImageJ and the total length of these lines is measured. The total VLD (mm/mm^2) is obtained by dividing the total vessel lengths by the area of all the measured squares.

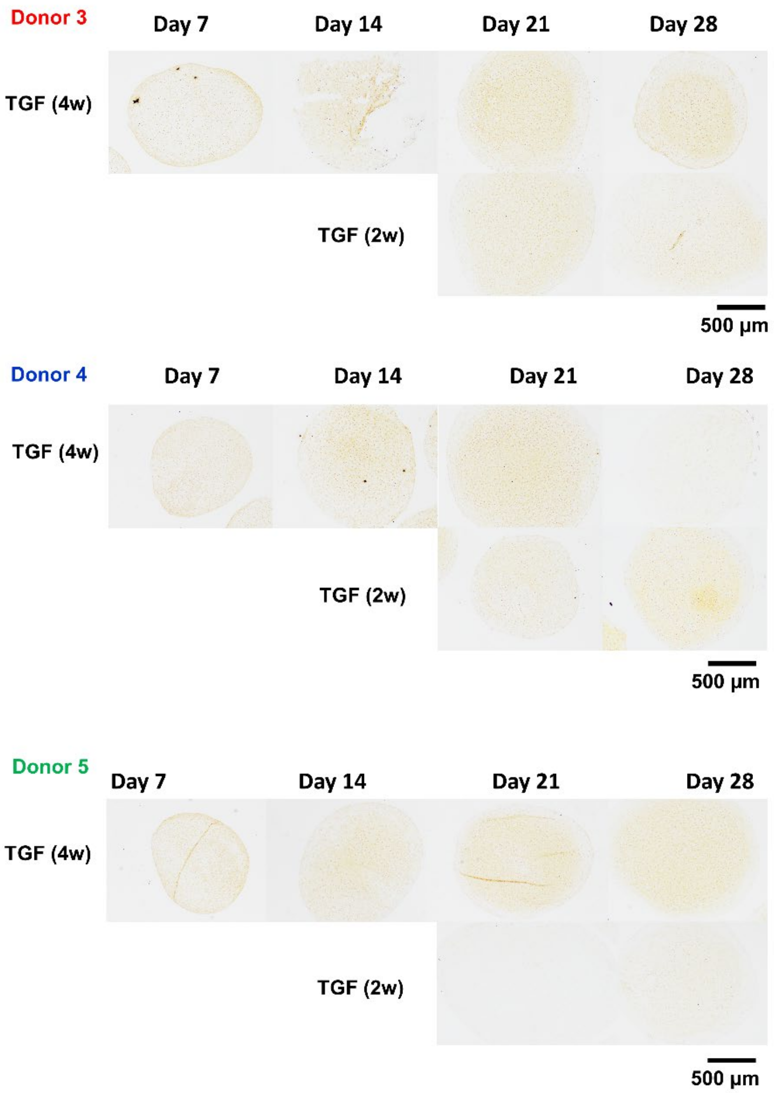

Figure S2. Von Kossa staining pellets cultured without BGP. Von Kossa staining of the groups TGF (4w) and TGF (2w) at day 7, 14, 21 and 28 of culture for donor 3, 4 and 5.

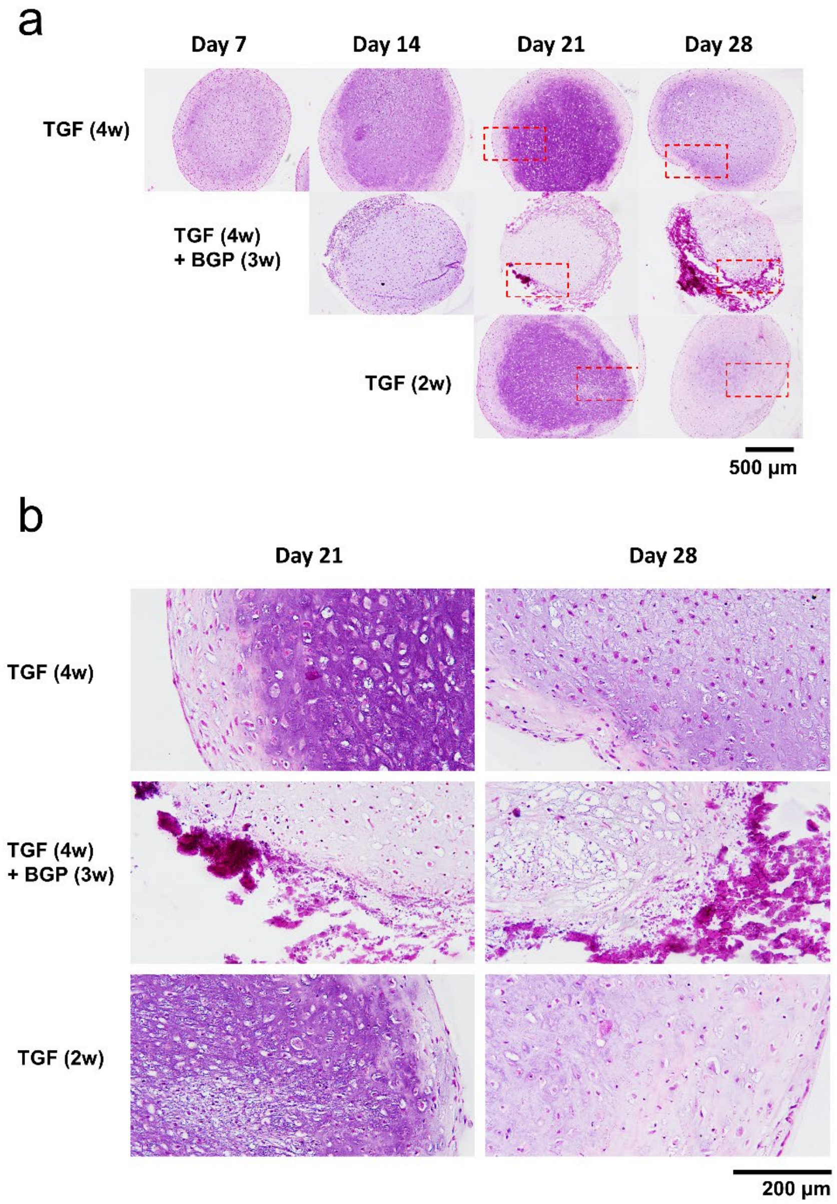

Figure S3. H&E stained histological sections. Representative H&E stained histological sections for the different experimental groups for donor 3. Images at low (**a**) and high (**b**) magnification are shown.

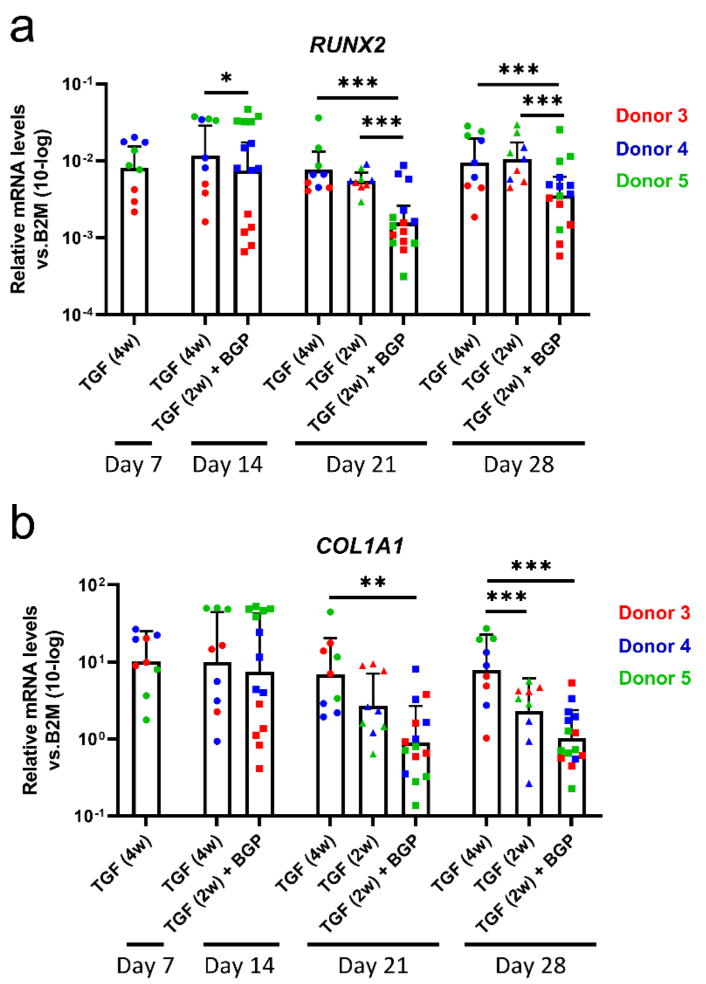

Figure S4. mRNA expression of RUNX2 and COL1A1. mRNA expression of RUNX2 (**a**) and COL1A1 (**b**) determined by qRT-PCR. B2M was used as the housekeeper gene. Data are presented as average ± SD (N=3 hMSC donors). *0.01< $p < 0.05$, **0.01< $p < 0.001$, ***$p < 0.001$.
